# Evaluating the Public's Interest in Testicle Tanning: Observational Study

**DOI:** 10.2196/39766

**Published:** 2022-09-12

**Authors:** Ryan Ottwell, Katherine Cox, Taylor Dobson, Muneeb Shah, Micah Hartwell

**Affiliations:** 1 Department of Dermatology St Joseph Mercy Hospital – Livingston Howell, MI United States; 2 Oklahoma State University Center for Health Sciences Tulsa, OK United States; 3 School of Nursing Oakland University Rochester, MI United States

**Keywords:** general dermatology, google trends, testicle tanning, UV radiation, public trends, skin cancer, cancer, harmful, internet, health trends, tanning

## Abstract

**Background:**

A new and potentially dangerous health trend, *testicle tanning*, received extensive media attention following a popular television program where a health and fitness influencer touted that testicular tanning increases testosterone levels. It has been shown that the public has a particular interest in tanning *wellness* trends; thus, given the vague nomenclature of the practice, the abundance of misleading information and support for using UV light by other health influencers may lead to an increase in men exposing themselves to UV radiation and developing associated complications.

**Objective:**

The aim of this paper is to evaluate the public’s interest in testicle tanning.

**Methods:**

Relative search interest was collected from Google Trends, and daily tweet volume was collected using Twitter via Sprout Social. The search was filtered to observe internet activity between February 1, 2022, and August 18, 2022. Autoregressive integrated moving average models were applied to forecast the predicted values through April 30 to compare to the actual observed values immediately following the airing of the show.

**Results:**

We found that the relative search interest for testicle tanning peaked (100) on April 19, 2022, following a discussion of the topic on a television program. Compared to the forecasted relative search interest of 1.36 (95% CI –3.29 to 6.01), had the topic not been discussed, it showed a 7252% increase in relative search interest. A similar spike was observed in the volume of tweets peaking on April 18 with 42,736. The expected number of tweets from the autoregressive integrated moving average model was 122 (95% CI –154 to 397), representing a 35,053% increase.

**Conclusions:**

Our results show that the promotion of testicle tanning generated significant public interest in an evidence-lacking and potentially dangerous health trend. Dermatologists and other health care professionals should be aware of these new viral health trends to best counsel patients and combat health misinformation.

## Introduction

“Testicle tanning” received extensive media attention following an episode of *Tucker Carlson Tonight*, where a health and fitness influencer touted that testicular tanning increases testosterone levels. While first described as exposing one’s scrotum to red-light therapy to enhance testosterone levels, this vague nomenclature and lack of supporting detail could mislead many into believing that exposure to UV light via sunlight or tanning beds will provide similar benefits. It has been shown that the public has a particular interest in tanning “wellness” trends [1]; thus, in this observational study, we evaluate the public’s interest in testicle tanning.

## Methods

Relative search interest (RSI; 0-100) was collected from Google Trends using the term “testicular tanning,” and from Twitter via Sprout Social (SproutSocial.com) using terms “testicular OR testicle OR ball OR balls OR scrotum” and “tan OR tanning OR sunning” to capture daily tweet volume. The search was filtered to observe internet activity between February 1, 2022, and August 18, 2022. Autoregressive integrated moving average models were applied to forecast the predicted values through April 30 to compare to the actual observed values immediately following the show’s airing [2]. Peak differences were calculated with 95% confidence intervals to estimate spikes in data.

## Results

**Figure 1:**
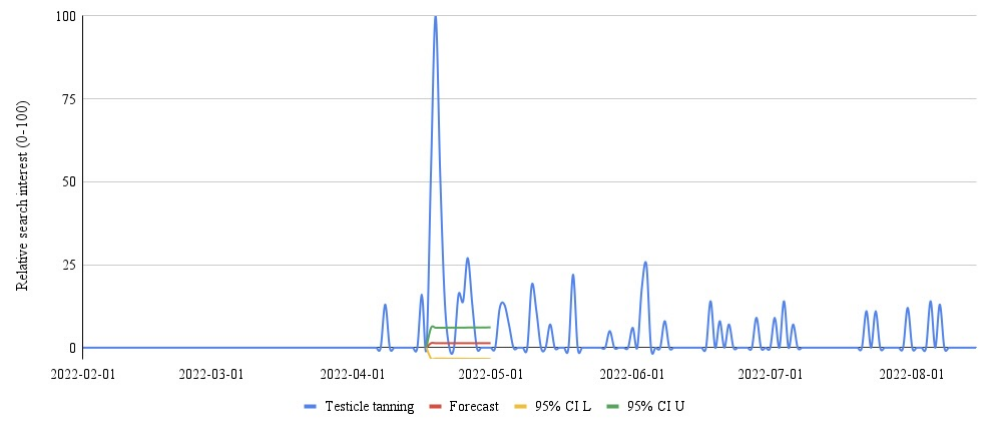
Search interest in "testicle tanning" from February 1, 2022, through August 18, 2022. L: lower; U: upper.

**Figure 2:**
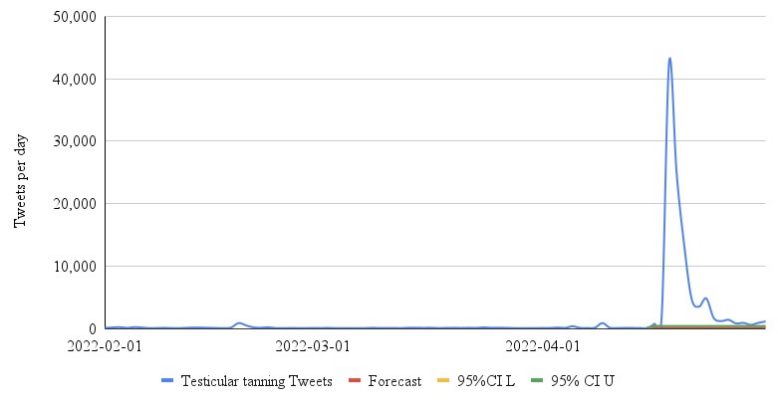
Daily number of tweets related to "testicle tanning" from February 1, 2022 to May 1, 2022. L: lower; U: upper.

We found that RSI for testicle tanning peaked (100) on April 19, 2022, following a discussion of the topic on the television program (Figure 1). Compared to the forecasted RSI of 1.36 (CI –3.29 to 6.01), had the topic not been discussed, this was a statistically significant difference, representing a 7252% increase in RSI. Continued search interest in testicular tanning was observed through August of 2022. A similar spike was observed in the volume of tweets peaking on April 18 with 42,736 (Figure 2). The expected number of Tweets from the autoregressive integrated moving average model was 122 (CI –154 to 397), a difference of 42,614, representing a 35,053% increase.

## Discussion

Similar to perineum sunning (a viral health trend performed by exposing one’s anogenital area to direct sunlight) [1], our results show that the promotion of testicle tanning on this television program generated significant public interest in an evidence-lacking and potentially dangerous health trend. The interest in this topic may be partially explained by the immense attention and advertising men’s sexual health and hormone replacement or hormone enhancing therapies receive in the US [3]. Although subsequent media coverage largely disfavored testicle tanning due to lacking evidence and potential dangers, other health influencers came to defend and encourage the practice of testicle tanning, specifically by using UV light [4]. Proponents of testicle tanning commonly cite a study from 1939, which found that in a small cohort of males all with “depressive mental states,” UV irradiation to the genitals increased urinary androsterone (a metabolite of testosterone) levels by “nearly 200%” [5]. Beyond this questionable study, research has shown that exposure to UV radiation may increase sex steroid hormone levels; however, these studies either do not include human participants or do not specifically evaluate UV radiation exposure to the genitals [6-8]. Research shows that excessive exposure to UV radiation may lead to higher rates of genital tumor formation and decreased sperm counts, as spermatogenesis is temperature dependent [9,10]. Thus, given the current obsession with optimizing male hormone levels, the high cost of red-light therapy, and misleading information and labeling of testicle tanning by prominent influencers, there may be an increase in men exposing themselves to UV radiation and developing associated complications. Limitations of our study include the retrospective cross-sectional design and the inability to determine the public’s intent, which necessitates future research.

Our study highlights how a non–scientifically based and potentially dangerous tanning practice can generate significant public interest. Similar to our findings, in a previous study published by JMIR Dermatology, it was found that public interest in perineum sunning continued after the initial social media post went viral (and continues to trend in social and news media stories nearly 3 years later); therefore, dermatologists and other health care professionals should be aware of these new viral health trends to best counsel patients and combat health misinformation.

## Abbreviations

RSI: relative search interest
